# Where are We Headed? Evidence to Inform Future Football Heading Guidelines

**DOI:** 10.1007/s40279-023-01852-x

**Published:** 2023-06-07

**Authors:** Kerry Peek, Rob Duffield, Ross Cairns, Mark Jones, Tim Meyer, Alan McCall, Vincent Oxenham

**Affiliations:** 1 Discipline of Physiotherapy, School of Health Sciences, Faculty of Medicine and Health, The University of Sydney, Sydney, NSW Australia; 2 School of Sport, Exercsie & Rehabilitation, Faculty of Health, University of Technology Sydney, Sydney, NSW Australia; 3 Football Australia, Sydney, NSW Australia; 4 Newcastle Sports Medicine, Warners Bay, NSW Australia; 5 School of Medicine and Public Health, The University of Newcastle, Newcastle, Australia; 6 School of Psychological Sciences, Faculty of Medicine, Health and Human Sciences, Macquarie University, Sydney, NSW Australia; 7 Department of Neuropsychology, Royal North Shore Hospital, Sydney, NSW Australia; 8 Institute of Sports and Preventive Medicine, Saarland University, Saarbrücken, Germany

## Abstract

Given the scientific and public concern regarding the short-, medium- and long-term consequences of heading on brain health, being proactive about developing and implementing guidelines that help reduce the burden (volume, impact magnitude and injury risk) of heading in young and beginner players appears justified. This narrative review explores the evidence underpinning strategies that could be incorporated into future heading guidelines to reduce heading burden in players across all levels of football. A four-step search strategy was utilised to identify all data-based papers related to heading in football. Eligibility criteria for inclusion were: (1) original data, (2) study population included football players, (3) outcome measures included one or more of the following: number of headers, measurement of head acceleration during heading, or head/brain injury incidence, and (4) published in English or English translation available. In total, 58 papers were included that outlined strategies based on (1) game or team development, (2) player skill development and (3) equipment. In particular, greater emphasis existed for small-sided games (particularly in young players) where fewer headers are observed when compared with the conventional 11 versus 11 game, as well as reducing headers from goal kicks and corners. Evidence also existed for developing a heading coaching framework that focusses on technical proficiency as well as neuromuscular neck exercises integrated into general injury reduction exercise programs, enforcement of rules related to deliberate head contact and using lower-pressure match and training balls. To mitigate potential risks of heading on brain health, a number of pragmatic strategies have been examined in scientific studies and may be considered as part of future heading guidelines.

## Key Points

**Table:** 

This review is the first to outline and summarise the current evidence and make recommendations for the inclusion of different strategies to assist football governing bodies worldwide when drafting and standardising heading guidelines to protect the long-term brain health of current and future generations of players.
It is also recommended that an implementation and evaluation plan which is co-designed by health professionals, researchers, coaches, players and other important stakeholders is developed in tandem to optimise the potential adherence to, and benefits from, any future heading guidelines.

## Introduction

Football is one of the very few sports where the head is purposefully used as an integral requirement of the game, with the skill of heading taught from a young playing age [1, 2]. The extent and effect of heading on players’ cognitive and emotional health is a key component of current discussions, with media, scientific and lay narratives arguing whether heading should be banned, curtailed or left unabated in football [1]. These conversations normally include two generic areas, (1) the effect of long-term repetition of heading on players’ brain health, and (2) what strategies exist to protect players from any potential health risks associated with heading [1, 3–5].

In regard to the effects of heading on brain health, review papers published to date report equivocal collective evidence of the causative relationship between repetitive heading and short- to medium-term cognitive changes [3, 5–12]. Although evidence exists for short-term (< 24 h) changes in biomarkers related to oculomotor function and neurofilament proteins following an acute bout of heading [13–15], these changes have been reported to be transient in nature, resolving as early as 24 h post heading exposure [13]. Further, the heading bouts often used in these studies were unrealistically high (e.g. 10–20 headers in 10 min) [13, 14] and not representative of game situations. The general consensus amongst these papers is that low heading exposure does not appear to impact cognitive skills in both youth and adults in the short term (i.e. < 72 h) [3, 5–12].

However, there is paucity of evidence regarding the long-term consequences of heading. A retrospective review of deceased former professional footballers from the Scottish League born before 1 January 1977 (age range 39–87 years, mean age 67.5 years), reported an increased likelihood of mortality from neurodegenerative conditions or being prescribed medications for dementia compared with a control population, with this effect only being apparent above the age of 70 [16]. Similar results were shown in a retrospective review of all-cause and cause-specific mortality in footballers who played at least one competitive match in France’s professional football championships between 1 January 1968 and 31 December 2015 [17]. The relationship of the specific act of heading to clinical or mortality outcomes in these studies is challenging given the lack of heading data for each player and the long time period between heading exposure and death [16, 17].

In studies where heading data were collected, the results are conflicting. One study of 60 retired professional players (mean age 67.5 years, range 39–87 years, average professional career length 15.7 years) [18] reported that lower scores on cognitive testing were associated with higher self-reported headers over a career. This finding is in contrast to an earlier study which reported no association between higher heading exposure over a career with lower cognitive skills or a higher risk of dementia [19]. The paucity of data and equivocal overall findings make it difficult to provide definitive conclusions on the causative effect of long-term heading exposure on brain health, which is likely to remain unchanged for the foreseeable future. Further, many limitations have been acknowledged, including the bias that may occur due to the use of subjective measures to quantify heading frequencies (e.g. self-report), not incorporating heading numbers during pre-season or training, only studying professional male cohorts, inability to generalise findings to amateur or recreational players, and self-selection bias, where only participants with perceived cognitive problems took part in the studies [16–18]. This has led to a recommendation that further longitudinal research be conducted to delineate current ambiguities of causal relationships [16, 17]. Despite the paucity of available high-quality research, many governing bodies are currently considering policy decisions on the appropriateness of heading in football; thus, further exploration of protective strategies is vital.

Given the scientific and public concern, but uncertainty regarding the short-, medium- and long-term consequences of heading on brain health, as well as the reported link between repetitive head impacts across all contact sports and chronic traumatic encephalopathy [20], there is a logical need to adopt a precautionary approach to mitigate any possible risks [21]. One such approach is being proactive about developing and implementing strategies, guidelines and frameworks that help reduce the burden (volume, impact magnitude, as well as injury risk) of heading until such a time that the evidence may be less equivocal. Although a limited number of Football Associations worldwide have banned or restricted the number of headers in young players [22, 23], most restrictions appear based on chronological age, rather than technical proficiency, physical, or biological determinants. For example, the first Football Associations to implement heading guidelines was US Soccer (USA), where heading is prohibited in players under 10 years and restricted in players aged 11–12 years. [24] In the USA, these guidelines were established in response to a 2014 class-action lawsuit by players and parents [24]. Given the understandable concerns for governing bodies, players and families, there is need for more refined understanding of available strategies to inform proactive development of future heading guidelines whilst research into the long-term effects of heading continues.

### Objective

The objective of this narrative review was to explore the current evidence underpinning pragmatic strategies that could be incorporated into future heading guidelines to reduce heading burden in players across all levels of football. Heading burden in this study relates to both the frequency of heading and resulting head impact magnitude (peak linear and rotational acceleration) of each header (as well as head/brain injury risk, where evidence exists) as potential risks to players’ long-term brain health.

## Methods

A narrative review methodology was intentionally chosen for this study to provide interpretation, critique and deeper understanding of the published literature on heading more broadly for policy makers, rather than address a specific research remit or narrowly focussed clinical question [25]. This choice of review enabled an iterative search process with an emphasis on snowballing and citation tracking to capture as many relevant papers as possible [26]. A four-step search strategy was developed to locate published literature on heading from the following electronic databases: PubMed, Web of Science, Embase (via Ovid) and SPORTDiscus (via Ebsco) from first inception to February 2022. First, a search was conducted by two authors using the search terms ‘soccer’ OR ‘football’ AND ‘head*’ for any published review papers. If a review paper was found, a retrieval of all the included studies in that review was completed. This was followed by a search of titles, abstracts and keywords for additional original data-based studies using the same search terms with full text screening. A review and retrieval of relevant papers within the reference lists of included studies was also completed. Eligibility criteria for the inclusion of data-based studies were (1) original data, (2) study population had to include football (soccer) players (studies which included football players as well as athletes from other sports were included, as long as the other criteria were met), (3) outcome measures must include one or more of the following: number of headers, measurement of head acceleration during heading, or head/brain injury incidence and (4) published in English or an English translation available. Studies were excluded from recommendations if they were based on non-human subjects such as mathematical, simulated or headform models (although these results may be discussed where relevant).

## Results

The search strategy was completed in February 2022, locating a total of *n* = 1918 papers, including *n* = 86 review papers and *n* = 1832 data-based studies, with 58 data-based studies included in this narrative review. Due to the heterogeneity of the included studies a narrative synthesis of the included studies is presented using the following framework of evidence relating to strategies based on:

1. Game or team development
2. Player skill development
3. Equipment

### Strategy 1: Game or Team Development Strategies

#### Heading Exposure

As context to the evidence of the importance of heading to the game, in the 2020 UEFA Men’s European Championships (2021), 25% of all goals scored were from a header [27], (as were 28% in the 2022 UEFA Women’s European Championships), rates similar to goals scored from headers in the 2002 FIFA Men’s World Cup (24%,) but more than the 2010 [28] and 2018 World Cups (18%) [29]. Furthermore, a study analysing goal scoring patterns of the Greek football league across seasons (2001–2002 to 2008–2009) reported that headers were the second most efficient goal scoring technique, representing 18.4% of the total goals scored, with kicks during free play being the most efficient method (67.1%) [30].

In terms of heading exposure, data from a recent systematic review [9], as well as two further studies in youth football [2, 31], showed the mean number of headers per player per game ranged from 0 to 8 headers in boys' football, 0–7 headers in girls' football, 2–11 headers in men’s football and 1–4 headers in women’s football. It is important to recognise that these mean heading data were calculated on the basis of the total number of headers per game (or per team) divided by the number of players. In reality there is rarely an even distribution of headers across all members of the team, particularly if the calculation includes goalkeepers (a position observed to infrequently head the ball) [2, 31, 32]. Thus, when considering heading burden, players who consistently head the ball more often in games may need to rest or reduce the number of headers in training to allow longer recovery between heading exposures. For example, it has been consistently shown that players in the central corridor, such as central defenders and midfielders, head the ball more frequently than players in other positions [2, 31], and this pattern of consistent heading in games occurs by ages 11–12 years old [2, 32, 33]. One concern with prohibiting heading in training, but not in games for young players who are yet to demonstrate heading technique proficiency, as observed in some heading guidelines [34], is that it is possible injury risk in young players may be increased due to poor technique development, timing, body positioning or using the side or top of their head to head the ball [21]. Consideration of individual player’s willingness to head the ball in games and the use of individual criteria, rather than team or age-based ones, may be more appropriate to determine when to commence heading technique training.

#### Head Impact Magnitude

It should be acknowledged that despite recent technological advances, it is currently unknown whether higher head impact magnitude measured using inertial measurement units corresponds to increased risk of stress and strain to brain tissue during heading [35]. It is also highly likely that linear forces will produce rotational and shear forces within the brain with finite element model-based biomechanical analyses of heading indicating that injury risk depends on many factors, including the head impact location, impact velocity and preparedness of the player [36]. Therefore the following head impact data should be interpreted with caution.

##### Age and Sex

Data pooled from studies in adult and youth players [37–50] demonstrated head impact magnitude (peak linear and rotational acceleration) during heading in:

- boys ranged from 9 to 45 g for peak linear acceleration and 501–10,372 rad/s^2^ for peak rotational acceleration.
- girls ranged from 5 to 47 g (peak linear acceleration) and 445–8869 rad/s^2^ for rotational acceleration.
- men 14–19 g (peak linear acceleration) and 656–774 rad/s^2^ for peak rotational acceleration.
- women 17–24 g (peak linear acceleration) and 1038–1416 rad/s^2^ for peak rotational acceleration.

In studies where head impact magnitude was directly compared between boys and girls, higher peak linear and rotational acceleration is often observed in girls, although this was not consistently shown across all studies [51, 52]. Peak linear and rotational acceleration during heading appear to decrease with age during adolescence [44], with maximum values for head acceleration during heading in adult players being consistently lower than youth players, and women often having higher values than men [37–50]. The reason for these observations require further elucidation, but is hypothesised to be related to the lower neck strength of women, adolescents and children, as well as differences in heading technique and experience, with emerging evidence to support this reasoning [50, 52].

##### Game Scenario

While scoring a goal is the ultimate aim in football, headers are also observed during different aspects of game play to either retain or gain possession of the ball, with varying head acceleration outcomes dependent on the delivery of the ball. Three studies have explored head acceleration measured during different types of headers during game play [38, 53] and a simulated environment [54]. A study of 25 women’s National Collegiate Athletic Association Division I players (mean age 19.6 years) reported that peak linear and rotational accelerations were the highest for headers following a goal kick (38.8 ± 19.4 g, 9.3 ± 3.9 krad/s^2^) or punt (36.0 ± 15.1 g, 10.1 ± 4.8 krad/s^2^) [53]. A further study of 36 elite female youth players, showed that on average, purposeful headers that occurred from shots on goal resulted in the largest peak linear head acceleration, while corner kicks resulted in the largest rotational velocity [38]. In terms of head impact location, headers that occurred on the top of the head resulted in the largest linear acceleration and rotational velocity, although most headers were performed by players using their forehead [38]. The third study, which explored the effect of three football header types (shooting, clearing and passing) and two heading approaches (standing and jumping) on impact forces and neck muscle activity, reported no difference in head acceleration between header types and approaches [54]. However, all balls in this study were delivered from a throw-in at 6.8 m/s which will influence these findings [54]. Interestingly, higher values of muscle activity were found for the right sternocleidomastoid for jumping versus standing headers, with the increased muscle activity observed during the jumping approach appearing to stabilise the head–neck complex on impact [54].

Hence, the context of the header is important to understand the likely heading burden. Limiting headers from goal and corner kicks in younger players, who are still learning proper heading technique, may limit cumulative linear and rotational head accelerations, in addition to reducing heading frequency [38, 53]. Encouraging young players to play out from the back, developing ball control with the chest or foot following long balls and encouraging short corners could limit the number of these headers, whilst also subscribing to many player development philosophies within a development curriculum. One simple way this could be accomplished is via constraints-based play such as small sided games (see 3.1.3.2 Constraints-Based Play below).

#### Heading Load

To date there have been very few published studies that have addressed the question of a ‘safe’ weekly heading load. The challenge in determining whether a ‘safe heading load’ exists is the heterogeneity between studies, including the various methods to quantify headers (e.g. self-report, observation, laboratory settings), inclusion or exclusion of training heading data and differences in the sensitivity, specificity and ecological validity of the cognitive measures used, as well as the uncertainty about the most appropriate target parameters to indicate ‘safety’. In addition, few studies report on players’ heading technique or make comparisons of field positional data or type of headers (e.g. headers from goal kicks, corners, crosses, throw ins). Most importantly, there are currently no studies which have prospectively collected heading data over the length of a playing career to assess changes in neurocognitive health over time. While neuropsychological/cognitive performance measures may help determine clinically meaningful changes in brain functioning, they are not the only measure which can explore whether a ‘safe’ heading load exists. Other studies using biochemical markers, blood biomarkers, imaging, visual reflexes, heart rate variability and transcranial magnetic stimulation may also be used to infer outcomes from different heading loads. However, insufficient evidence exists based on any of these measures to inform a ‘safe’ heading load, probably due to the lack of methodological standardisation and incongruent results between studies preventing clear conclusions regarding the extent of acute soccer heading risk, especially longitudinally [55]. Table 1 summarises the included studies (*n* = 11) which focussed on comparing neuropsychological/cognitive performance where no/lower heading load has been compared with higher heading load in football players separated by age group (youth and adult).

**Table 1:** Summary of heading load and neuropsychological/cognitive performance for youth and adult players

Study	Participants	Heading quantification method	Mean headers per player per game	Outcome measures used	Results
*Youth players*					
Stephens et al. (2005) [56]	*n* = 22 (all men) Age range: 13–16 years	Observation and self-reported	2.44 ± 1.89	Wisconsin Card Sorting Test, Stroop Test, Wechsler Adult Intelligence Scale – Revised Digit Symbol and Digit Span, Trail Making Test A and B, Rey Complex Figure, Wechsler Memory Scale Revised – Logical Memory I and II, Alternate Uses Test, and Computerised Test of Attention Performance – alertness, divided attention, covert attention shift, flexibility and working memory	Findings indicate the absence of neuropsychological impairment arising due to cumulative mild head injury incidence, or cumulative heading
Kaminski et al. (2007) [59]	*n* = 26 (all girls) Age range: 14–18 years	Counted during games by trainers	0.8 ± 1.2	Hopkins Verbal Learning Test, Wechsler Adult Intelligence Scale – III Digit Span Test	In high-school players there were no significant correlations between the total number of match headers and the change in scores for each of the outcome measures
Kaminski et al. (2008) [58]	*n* = 393 (all girls) Mean age: 15.8 ± 1.2 (range: 13–18 years)	Counted during games by trainers	1.0 ± 1.3	Simple reaction time, matching to sample, continuous performance test, math processing, and Sternberg memory	No detrimental relationship between the number of purposeful headers and neurocognitive measures
Stephens et al. (2010) [57]	*n* = 48 (all boys) Age range: 13–16 years	Self-reported	4.1 ± 3.0 (range 0–11)	Wisconsin Card Sorting Test, Stroop Test, Wechsler Adult Intelligence Scale – Revised Digit Symbol and Digit Span, Trail Making Test A and B, Rey Complex Figure, Wechsler Memory Scale Revised – Logical Memory I and II, Alternate Uses Test, and Computerised Test of Attention Performance – alertness, divided attention, covert attention shift, flexibility and working memory	Cumulative heading did not predict neuropsychological performance
*Adult Players*					
Witol et al. (2003) [60]	*n* = 60 (all men) Age range: 20–22 years	Self-reported	Players were divided into four groups: no heading (control), low (0–4 headers/ game), moderate (5–8 headers) and high (9+ headers)	Six cognitive tests: The Shipley Institute of Living Scale, Trail Making Test Parts A and B, Paced Auditory Serial Addition Test, Facial Recognition Test, Rey–Osterreith Complex Figure Test and Rey Auditory Verbal Learning Test	Players with the highest lifetime estimates of heading had poorer scores on scales measuring attention, concentration, cognitive flexibility, and general intellectual functioning. No significant differences were found for all groups when compared with the tests’ normative sample. Players’ current level of heading was less predictive of neuro-cognitive changes
Kaminski et al. (2007) [59]	*n* = 21 (all women) Mean age 19.1 ± 1.1 years (range 19–24 years)	Counted during games by trainers	2.7 ± 2.3	Hopkins Verbal Learning Test, Wechsler Adult Intelligence Scale – III Digit Span Test	No significant correlations existed between the total number of match headers per player for a soccer season and the change in scores on the battery of postural stability and neuropsychological tests
Rutherford et al. (2009) [62]	*n* = 25 (all men) Mean age: 20.4 ± 0.9 years	Self-reported	6.38 ± 4.41	Wisconsin Card Sorting Test, Stroop Test, Wechsler Adult Intelligence Scale – Revised Digit Symbol and Digit Span, Trail Making Test A and B, Rey Complex Figure, Wechsler Memory Scale Revised – Logical Memory I and II and Verbal Paired Associates I and II, Alternate Uses Test, FAS-Controlled Oral Word Association Test and Computerised Test of Attention Performance – alertness, divided attention, visual search, working memory, flexibility and covert attention shift	No evidence that neuropsychological test performance was related to the estimated accumulative number of headers
Stewart et al. (2018) [63]	*n* = 308 (78% men) Age range: 18–29+ years	Self-reported (headcount)	39.9–44.9 headers per player per 2-week period, including games and practice sessions	Groton Maze Chase Task and Cogstate Subtests: verbal learning, verbal memory, psychomotor speed, attention and working memory	Level of variation in neuropsychological function did not reach the level of clinical impairment. However, comparing low levels of heading versus higher levels of heading, the latter was associated with transient poorer performances on cognitive tasks that emphasized psychomotor speed, attention and working memory. All measures remained within the normal range compared to the tests’ normative sample
Levitch et al. (2018) [61]	*n* = 311 (83% men) Mean age: 26 ± 7.7 years	Self-reported (headcount)	34.17 headers per 2-week period, including training and games	Groton Maze Chase Task and Cogstate subtests: verbal learning, verbal memory, psychomotor speed, attention and working memory	Transient changes in cognition existed with heading, though were not severe enough to indicate clinical impairment. However, players reporting greater amounts of recent heading and long-term heading exhibited worse neuropsychological function in different neuropsychological domains
Kaminski et al. (2019) [65]	*n* = 87 (all female) Mean age: 18.9 ± 0.9 years	Artificial setting in lab: two groups – control simulated heading (no head-ball contact) and three heading groups	Heading groups completed 15 headers in 15 min repeated across two sessions with different types of headers	Self-reported concussion-related symptoms, and neurocognitive performance (ImPACT)	No decrements in neurocognitive performance. Although concussion-related symptoms were elevated, even at baseline, and continued to rise following heading drills in those with concussion histories
Amitay et al (2020) [67]	*n* = 159 (all adult, professional men) Specific age not stated	Headers counted during games using video analysis	Players were divided into two main groups: players with high heading exposure and players with low heading exposure, according to the median heading exposure per hour of game (1.34) Mean headers per season = 48.2	Rivermead Post-Concussion Syndrome Questionnaire (RPQ): 16 post-concussion symptoms including headaches, dizziness, nausea/vomiting, noise sensitivity, sleep disturbance, fatigue, irritability, feeling depressed/tearful, feeling frustrated/impatient, forgetfulness, poor concentration, taking longer to think, blurred vision, light sensitivity, double vision and restlessness, and Beck Depression Inventory and Mini Sleep Questionnaire	Higher heading exposure was associated with lower severity of post-concussion symptoms, depression symptoms, anxiety and sleep disorders
Strauss et al. (2021) [64]	*n* = 246 Mean age 25.48 ± 7.2 years (range 18–55 years) (plus 118 controls)	Self-reported (headcount)	mean = 37.9 heads/2-week period including training and games	Groton Maze Chase Task and Cogstate subtests: verbal learning, verbal memory, psychomotor speed, attention and working memory	Athletes with no or lower exposure to heading demonstrated better cognitive performance compared with non-athletes. Players with the highest exposure to heading did not differ significantly from healthy non-athletes on cognitive performance

Youth players: in youth players aged 13–18 years, four studies [56–59] have shown that under four headers per game did not result in significant changes in cognitive performance in the short- to medium-term, over one to three seasons (Table 1). These studies used a variety of measures, including comprehensive neuropsychological batteries or computerised cognitive tools (e.g. ImPACT). Two studies in boys aged 13–16 years which administered 13 cognitive tests reported that cumulative heading was not associated with cognitive performance [56, 57]. Further, no detrimental relationship was observed between the number of purposeful headers and cognitive measures in two studies of girls aged 12–18 years. [58, 59] In these four studies the mean number of headers per player per game ranged from one to four, indicating that low number of headers per game may represent a heading load which does not lead to short- to medium-term changes on cognitive testing. However, these results may not generalise to players who perform higher numbers of headers per game, as well as during training, nor do they indicate what the long-term changes may or may not be if these players continue to head the ball over a 10–20 year playing career.

Adult players: in adult players, aged 18–55 years, seven studies [58, 60–65] explored the short- to medium-impact of heading on cognitive performance with the mixed and equivocal findings outlined in Table 1. Four studies reported no relationship between higher number of headers and cognitive performance [59, 62, 65, 66], whereas three studies. [60, 61, 63] reported an inverse relationship in that higher heading load resulted in reduced cognitive performance, though this reduction did not meet the threshold representing clinical impairment. Further, the cognitive performance of athletes in the high heading load group remained within the normal range for their age. [17, 23, 24] A further study of 159 male professional players from the Israeli Premier League reported that higher heading exposure was associated with lower severity of post-concussion symptoms, depression symptoms, anxiety and sleep disorders [67]. There are several methodological challenges when interpreting differences in study findings, as not all of these studies included heading data from training, with most relying on self-report [60, 62]. Thus, more research is needed before quantification of a maximum weekly or sessional heading load which could be considered ‘safe’ can be confidently made.

Applying a weekly or sessional heading load threshold would be one of the most convenient safety modifications to the game; however, current research on the short- to medium-term impact of heading on player brain health remains ambiguous and any threshold is likely to be an arbitrary value. However, given the uncertainty [19] but potential for long-term adverse effects of heading [16, 18, 68, 69], it seems sensible to reduce heading burden by removing from training any unnecessary heading drills that do not translate to game play. An additional approach could be to limit the number of headers per player per week in training to what is observed in games (with maximum mean values ranging from 8 to 11 headers per game in boys' and men’s football and 4 to 7 headers per game for girls and women) [9], with similar periods of rest between headers during training drills (most headers in games will occur over a 60–90 min period depending on the age of players). However, it is acknowledged that this strategy raises the practical problem of how to count heading frequency in games and whose responsibility this is, particularly in under-resourced clubs within the broader football playing community.

##### Rest and Recovery from Heading

Given that any acute effects observed (such as changes in biomarkers and neurocognitive testing) in players following a single bout of heading usually resolves within 72 h [13–15], consideration could also be given towards scheduling rest and recovery periods from heading. This might include resting players from heading drills in training for the week following a game in which they have experienced higher than average heading loads.

##### Constraints-Based Play

Constraints-based play represents alterations to the rules and playing conditions with a view to altering player engagement with the ball or opponents. Small-sided games (SSG), also known as game-based training or skill-based conditioning games, are games played with adapted rules, such as using reduced pitch size along with fewer players per side [70]. SSG are often used in football due to their multifunctional nature, such as to stimulate an increased intensity compared with a full match, while subsequently developing specific tactical skills [71]. The use of SSGs increases the frequency of skills such as dribbling, tackling, crosses and shots on goal [70–72], since reduced spaces results in closer opponents and increased speed of technical execution [70]. In contrast, the use of a large field results in more opportunity to retain possession of the ball, perform longer passes, participate in more aerial play and in turn perform more headers [70]. Given the type, context and magnitude of headers reported earlier, greater use of SSGs, particularly in young players during training, may lessen heading burden by reducing the necessity to head the ball, or at the very least reduces headers from long-range balls. Table 2 summarises the literature related to heading frequency using different game formats and may present a method to reduce exposure to heading by virtue of altered game playing format within smaller, age-appropriate pitch dimensions while also transitioning young players to the competitive 11 versus 11 format on a full-sized pitch in an older age group.

**Table 2:** The effect of changes to game format on heading frequency

Study	Participants	Methods – format of small sided games	Results – heading frequency (mean ± standard deviation)	Study conclusion
Martone et al. (2017) [73]	*n* = 17 males (U12), mean age: 10.0 ± 0.5 years *n* = 16 males (U14), mean age: 13.2 ± 0.3 years	6 × (3 × 4 min various SSG formats)	U12 range: 0.3 ± 0.0–1.0 ± 0.0 per player per 12 min (depending on pitch size and area per player) U14 range: 0.3 ± 0.6–0.7 ± 1.2 per player per 12 min (depending on pitch size and area per player)	Headers per player increased from 0.3 to 1.0 with increasing pitch area from 40 m^2^ to 90 m^2^ in U12, but remained between 0.3 and 0.7 headers/player across all area sizes in U14s
Rago et al. (2016) [74]	*n* = 8 males, mean age: 23.6 ± 2.3 years, semi-professional players	3 × 6 min, 5 versus 5 SSG	0.1 ± 0.3 headers per player per 18 min	Headers remained very low in this SSG format
Owen et al. (2014) [75]	*n* = 16 males, mean age: 27.6 ± 4.1 years, professional players	16 × (3 × 5 min, various SSG, MSG and LSG formats)	SSG (4 versus 4): 3 ± 2 per 15 min MSG (5 versus 5 to 8 versus 8): 4 ± 1 per 15 min LSG (9 versus 9 to 11 versus 11): 9 ± 2 per 15 min	In formats with low number of players, more passes, dribbling and shots, but fewer headers were observed
Owen et al. (2011) [76]	*n* = 15 males, mean age: 26.3 ± 4.9 years, professional players	SSG 3 versus 3 plus goalkeepers, LSG 9 versus 9 plus goalkeepers lasting for 3 × 5 min with 4-min passive recovery	SSG 6 ± 1 per player LSG 15 ± 1 per player	More headers were performed in LSG when compared with SSG (effect size 15.8, *p* ≤ 0.05)
Da Silva et al. (2011) [77]	*n* = 16 males, mean age: 13.5 ± 0.7 years, professional players	One season, exact exposure unknown	3 versus 3 SSG: 1 ± 1 per player per 12 min 4 versus 4 SSG: 1 ± 1 per player per 12 min 5 versus 5 SSG: 1 ± 1 per player per 12 min	The number of players had no effect on the number of headers per player, which remained low for all SSG formats
Casamichana et al. (2010) [78]	*n* = 10 players, mean age: 15.5 ± 0.5 years	5 versus 5 plus goalkeeper (32 × 23 m, 50 × 35 m, 62 × 44 m)	1.7 ± 1.0 per player, 32 × 23 m 2.3 ± 2.2 per player, 50 × 35 m 4.0 ± 2.1 per player, 62 × 44 m	The mean number of headers performed per player per game increased with increasing pitch size
Katis and Kellis (2009) [79]	*n* = 34 players, mean age: 13 ± 0.9 years, amateur players	3 versus 3, 6 versus 6 (10 × 4 min duration with 3 min active recovery)	3 versus 3: < 2 per player 6 versus 6: < 5 per player	Fewer headers were performed in 3 versus 3 games than 6 versus 6 games (*p* ≤ 0.05, likely related to the increased number of long passes in 6 versus 6 games

*SSG* small sided games, *MSG* medium sided games, *LSG* large sided games, *U* under

##### Rule Enforcement

In 2006, following analyses of head injury mechanisms during the FIFA World Cups (1998–2004), the International Football Association Board (IFAB) changed the laws of the game so that deliberate elbow-head contacts resulted in the offending player being permanently removed from play [80]. This rule change led to a 29% reduction in head injuries in a national league [81]. Acknowledgement and enforcement of this rule should be considered important at all levels of the game.

**Summary of Game or Team Recommendations for Future Heading Guidelines**

- Monitoring heading exposure in both games and training for all players is recommended, with a view to resting players who regularly head the ball in games from heading in training the following week, particularly heading drills with balls delivered via goal or corner kicks. This should be emphasised for younger players (who may be less able than adults to make an informed decision based on the possible long-term risks associated with heading) as well as girls and women, as these players may be exposed to higher head accelerations during heading than adult men.
- Coaching frameworks should focus on attaining heading proficiency which includes teaching players to receive the ball on their forehead within a controlled environment before players head the ball in games (many elements of heading training can occur without ball–head contact).
- Greater emphasis on SSG, playing out from the back, playing short corners, and discouraging headers from goal kicks should be considered as a strategy to reduce heading burden, particularly in young developing players.
- Rules concerning deliberate head contact in games must be enforced.

### Strategy 2: Player Skill Development Strategies

Strategies that relate to individual players such as playing position, level of experience, heading technique proficiency and neck strength are considered in this section.

#### Playing Position, Experience and Heading Technique

The evidence for the influence of player characteristics, including playing position, experience and heading technique on head acceleration during heading is presented in Table 3. Evidence supports that head acceleration during heading can be reduced by improved technique [82], such as using the forehead to head the ball [38, 42] and improved body positioning [83, 84]. This supports the notion that teaching heading technique should be considered an important and integral strategy for any future heading guidelines. Hence, consideration of the content, age and education processes of technical instruction within the coaching curriculum of a football organisation will be an important strategy to develop.

**Table 3:** Influence of player characteristics on head acceleration during heading

Study	Participants	Methods	Sensor type	Results	Study conclusion
*Playing position*					
Erkman (2009) [85]	*n* = 243 professional adult players, mean age: not stated	The assessment of accuracy and coordination in heading a ball of professional football players of different positions using the FIFA Medical Assessment and Research Center (F-MARC) test battery.The test awarded a maximum of 12 points depending on where the headed ball hit the goal from two different thrown ball deliveries (middle of the goal and from the right side of the goal post)	N/A	The mean (SD) score for headers following balls thrown from middle of the goal for: goalkeepers = 6.94 ± 3.38, defenders = 8.47 ± 4.35, midfielders = 8.26 ± 3.53, forwards = 9.41 ± 3.31 The mean (SD) score for headers following balls thrown from right side of the goalpost for: goalkeepers = 4.32 ± 3.15, defenders = 6.13 ± 3.45, midfielders = 5.68 ± 3.77, forwards = 8.53 ± 3.48	Goalkeepers were less talented than defenders, midfielders and forwards at heading a ball thrown from middle of the goal. Conversely, the most talented players at heading balls thrown from the right side of the goalpost were forwards, followed by defenders and midfielders
*Experience*					
Worsey et al. (2020) [82]	*n* = 8 (all men, different skill level), mean age: not stated	Calculation of the acceleration ratio as an attenuation index for players of different skill levels during a front heading activity (with a lower ratio representing less head acceleration in relation to torso acceleration)	IMU and 3D motion capture with full body markers	For novice participants, the ratio was as high as 8.3 (mean value 5.0 ± 1.8), whereas, for experienced players, the mean ratio was 3.2 ± 1.5. Elite players stiffen the neck muscles to increase the ball velocity and so the torso acts as a support structure. Electromyography (EMG) signals that were recorded from the neck and shoulder before and after a training intervention showed a major increase in mean average muscle activity (146%, *p* = 3.39 × 10^−6^). This was accompanied by a major decrease in acceleration ratio (34.41%, *p* = 0.008). The average head–ball impact velocity was 1.95 ± 0.53 m/s, determined while using optical motion capture. For this low velocity, the impact force was 102 ± 19 N, 13% of the published concussive force	The voluntary action of neck muscles decreases isolated head movements during heading. A comparison between the acceleration impacts of heading the ball and player experience has demonstrated a significant difference in the preparation for and accommodation of the impact. The ratio between the maximum acceleration of the head and the maximum acceleration of the torso represented by an accelerometer located at the T3 vertebra indicates a failure of novice players to adequately prepare for the impact. Coaches and trainers may use this evidence in their development of junior players
Hernandez and Chen-Hua (2016) [86]	*n* = 6 adult men (3 less proficient and 3 experienced players), mean age: not stated	Motion capture analysis of heading technique within a laboratory setting	Motion capture with full-body markers	For upper body kinematics, the proficient subjects exerted lesser elbow angles [47.8" (1.9)] than the less proficient [58.7"(3.5)]. In the case of lower body kinetics, the proficient subjects exerted greater ankle moment [1.9(O.2) Nm/Kg] than less-proficient subjects [1.5(0.3)Nm/Kg]	Experienced players generally exhibited more desirable characteristics during each heading trial, such as better joint coordination and higher ground impulse, representing an overall greater ability in implementing the skills
*Technique*					
Wahlquist and Kaminski (2021) [87]	*n* = 12 girls, mean age: not stated, but all under 12 years	Girls wore a head band sensor during two bouts of heading, using a lightweight ball, one before and one after completion of the Get aHEAD Safely in Soccer™ program intervention. Participants completed balance (BESS and SWAY) and neurocognitive function (ImPACT) tests at baseline and after each bout of heading	Instrumented headband	There were no significant changes in head impact biomechanics, BESS or ImPACT scores pre- to post-season. Deficits in three of the five SWAY positions were observed from baseline to post-season	Following the intervention, the coaches and researchers observed an improvement in heading technique/form, although this was based on subjective assessment only
Quintero et al. (2020) [88]	*n* = 3 girls aged 12 years	Researchers implemented a 10-week behavioural skills training program for heading. Heading practice sessions with a size 4 foam ball were recorded to visually analyse the slow-motion video to score each trial to assess performance using a 14-item checklist	Video analysis	Each participant demonstrated an overall increase in the percentage of correct steps completed from baseline to training	Results support the use of behavioural skills training as an effective method to improve correct heading technique, as evidenced by an overall increase in the percentage of correct steps for executing a correct header based on the 14-step task analysis. A larger sample size is required to assess these effects in a more varied cohort of players
Harriss et al. (2019) [38]	*n* = 36 girls, mean age: 13.4 years	Elite youth football teams (U13, U14, U15) were followed for an entire football season. Players wore wireless sensors during each game to quantify head impact magnitudes. A total of 60 regular season games (20 games per team) were video recorded, and purposeful heading events were categorized by game scenario (e.g. throw in) and head impact location (e.g. front of head)	Instrumented headband	The rotational head velocity from purposeful headers varied significantly between head impact locations (*χ* [2] = 18.15, *p* = 0.0001). Purposeful headers that occurred at the top of the head resulted in larger rotational velocities compared with the front of the head [*t*(429.49) = 4.30, *p* = 0.0001]. There was no statistically significant difference in rotational velocity between purposeful headers that occurred at the front of the head compared with the side of the head [*t*(430.35) = 0.54, *p* = 0.59]	While most headers were performed using the front of the head, players still use the top of their head for almost one-third of purposeful headers. Rotational head velocity was larger for headers performed with the top of the head compared with the front of the head
Huber et al. (2019) [42]	*n* = 23 boys, *n* = 18 girls, mean age: not stated but in high school grades 9–12	Headers coded from match play where players were instrumented with a headband-based head impact sensor composed of a triaxial accelerometer and gyroscope with headers verified via video analysis	Instrumented headband and video analysis	Female player-to-player impacts (6.2 ± 3.8 rad/s2) resulted in significantly higher peak angular accelerations than males (4.3 ± 3.0 rad/s2; *p* = 0.026). Male (17.6 ± 6.6 rad/s) and female (16.6 ± 7.1 rad/s) frontal head-to-ball impacts resulted in significantly lower mean peak angular velocity than side (*M* = 21.6 ± 7.2 rad/s, *p* < 0.001; *F* = 22.4 ± 9.1 rad/s, *p* = 0.041) and rear (*M* = 23.6 ± 8.2 rad/s, *p* < 0.001) or crown (*M* = 20.3 ± 6.8 rad/s, *p* = 0.001; *F* = 20.8 ± 8.2 rad/s, *p* = 0.003) head-to-ball impacts	Proper heading technique using the front of the head should be emphasised to reduce kinematic loading in repetitive heading
Gallant et al. (2017) [83]	*n* = 51 adult players, mean age: not stated,	players were (*n* = 25) or were not (*n* = 26) given training in proper heading after which they were tested on a head-ball task. The serial reaction time test (SRTT) was administered before (pre-test) and after (post-test) the training and testing	N/A	Improper heading did not disrupt SRTT performance with both groups demonstrating a decrease in reaction time from pre to post test	There was some evidence of implicit learning on the pre-test and clear evidence of continued implicit learning on the post-test. Participants who had been trained in proper techniques did head the ball better than those who had not (using a heading checklist)
Ludwig (2001) [84]	*n* = 24 women’s collegiate football players with at least 9 years of football heading experience	Players were separated into frequent heading (FH) and infrequent heading (IH) groups. Ten maximal standing headers were videotaped and analysed	Video analysis	The frequent header group (FH) added significantly more velocity to the ball post-contact than the infrequent header group (IH). The FH group had significantly larger change in trunk ROM and neck and trunk angular velocity at head-to-ball contact	Female players who head the ball frequently use a different technique than players who head the ball infrequently. The difference in technique between FH and IH was manifested in a difference in ball velocity from pre-contact to post-contact. The variables responsible for this change in velocity are: (a) increased ROM and angular velocity of the trunk, and (b) a short, powerful flexion of the neck beginning just prior to contact and continuing through contact. This change in velocity of the ball could be a very good predictor of experienced heading technique

*IMU* inertial measurement unit, *EMG* electromyography, *BESS* balance error scoring system, *SWAY* balance test, *ImPACT* neurocognitive function tests, *SRTT* serial reaction time test, *FH* frequent heading, *IH* infrequent heading groups, *ROM* range of movement

#### Neck Muscle Training to Reduce Head Acceleration and Injury Risk

A recent systematic review [51] presented evidence in support of a relationship between higher maximal isometric neck strength and lower head impact magnitude during purposeful heading in football. This apparent relationship has provided the rationale for the investigation of neck muscle training interventions and their effect on head acceleration during heading and/or heading-related injury outcomes (Table 4). Specifically, intervention studies which demonstrated a reduction in head acceleration [52, 89] or injury risk [117] associated with heading included both neck and general exercises as part of a neuromuscular training program [52, 89, 117]. In contrast, intervention studies that did not demonstrate reduced head acceleration employed isometric (using peer or self-resisted exercises) or isotonic neck exercises [45, 87]. These findings may indicate the need for increased specificity of neck training programs that relate specifically to the requirements of heading to be integrated into a more generalised neuromuscular program, such as the FIFA 11+ or within an organisation’s internal programs i.e. Football Australia’s Perform+. [52, 117] Further, results from a recent systematic review [90] demonstrated limited evidence (*n* = 6 studies) that greater neck strength and/or participation in injury reduction exercise programs, which included neck exercises, may reduce the incidence of sport-related head and neck injuries or concussion in contact sport athletes more broadly. Within this review paper there were three studies (summarised in Table 4) that included football players amongst a range of other athletes involved in basketball, American Football, lacrosse and volleyball (thus results for football players cannot be distinguished from other sports) [91–93]. The results showed a lower incidence of concussion in athletes with higher maximal isometric neck strength (but not deep neck flexor endurance) and for athletes who completed training programs which included specific neck exercises [90]. Finally, the only known study to explore the relationship between neck strength and psychological distress in collegiate football players (*n* = 29) and limited/non-contact sport athletes (*n* = 63) reported that higher neck strength in football players was associated with better scores on the anxiety subscale of the Brief Symptom Inventory-18 (*p* = 0.02) which was not observed in limited/non-contact athletes [106]. Recent work from the same authors’ group that included a sample of the same participants in both studies demonstrated that greater neck strength was associated with more intact white matter organisation in football players [94]. These findings appear consistent with earlier published literature suggesting that the neck musculature plays an important role in limiting the transfer of force to the brain by decreasing linear and rotational head accelerations that occur during heading [47, 52, 94, 95]. This may in turn reduce the potential for neural damage, although further research is needed to explore this further [95]. In addition, when neck flexor strength was weaker than neck extensor strength and right rotator strength was weaker than left rotator strength, football players reported more somatisation symptoms, but fewer depression symptoms, with no significant associations noted in limited/non-contact athletes [106]. These results support that neck muscle strength imbalances may also play an important role in stabilising the head on contact [95–99].

**Table 4:** The effect of a neck training intervention on reducing head acceleration and injury incidence in athletic populations which include football players

Study	Participants	Intervention	Results	Study conclusions
*Head acceleration during heading*				
Peek et al. (2022) [52]	*n* = 52 girls and boys (*n* = 31 intervention, *n* = 21 control), mean age: 14.65 years	Explored the effect of a neuromuscular neck exercise program on (a) neck strength and (b) head impact magnitude during heading in boys and girls. Players were randomised by team to the intervention (5-week supervised neuromuscular neck exercises integrated into part 2 of FIFA 11+, completed × 3 per week) or control (FIFA 11+, but no neck exercises)	Significant differences were observed in neck strength variables (*p* < 0.001) and peak linear acceleration (*p* < 0.01, *η* [2] = 0.14) between the intervention and control groups over time. Intervention players demonstrated increases in mean composite neck strength (53.8% intervention versus 15.6% control), as well as decreases in mean peak linear head acceleration during heading (−11.8% versus −5.0%) from baseline to follow-up. Reduction in peak angular velocity was more pronounced in girls (−27.7%) than boys (−11.5%) in the intervention group	Players who completed neck exercises demonstrated an increase in isometric neck strength and decrease in head acceleration during heading. These exercises were easily incorporated into usual training, taking less than 2 min to complete per session
Wahlquist et al. (2021) [87]	*n* = 12 girls, mean age: 10.5 years	Get aHead Safely in Soccer program completed × 2 per week over one season which included core and neck exercises (manually resisted isometric neck flexors, extensors and side-flexors)	There were no changes in peak linear acceleration, peak rotational acceleration, or peak rotational velocity from pre- to post-season heading sessions	Although there were no changes in head impact kinematics, coaches and researchers noted an improvement in heading technique
Muller et al. (2020) [99]	*n* = 27 boys and girls (*n* = 14 intervention), age range: 15–18 years	Twice weekly neck exercises incorporated into a neuromuscular exercise program for 14 weeks	Female athletes had lower isolated neck strength (*p* ≤ 0.004), lower functional neck strength (*p* ≤ 0.017) and higher total peak linear acceleration during purposeful headers compared with males (17.2 ± 3.5 g and 13.0 ± 2.3 g, respectively, at 9.6 m/s ball velocity during impact; *p* = 0.003). The intervention group showed moderate to large strength gains resulting in lower peak linear acceleration during heading	Resistance training focussing on cervical and trunk musculature is practicable in youth football, elicits strength gains and helps to mitigate peak linear acceleration during purposeful heading. Results should encourage youth strength and conditioning professionals to incorporate neck exercises as a risk reduction strategy into their training routine
Becker et al. (2019) [45]	*n* = 33 men, mean age: 20.3 ± 3.6 years	Six-week strength training program of the neck flexors and extensors	No significant change of isometric neck strength over time between the groups (*F* = 2.265, *p* = 0.121). Head acceleration was not reduced significantly for standing (*F* = 0.796, *p* = 0.460), jumping (*F* = 1.272, *p* = 0.295) and running (*F* = 1.050, *p* = 0.362) headers	The predicted preventive benefit of a 6-week strength training program of neck flexors and neck extensors could not be confirmed statistically in this study
*Injury incidence*				
Morrissey et al. (2019) [93]	*n* = 119 high school American Football, football and volleyball athletes (70 boys, 49 girls), mean age: not specified	Pre-experimental study on the use of core training programs which included neck exercises as a preventative tool for concussion	Concussion diagnosed using the Westmont Hilltop School District criteria. Expected number of concussions for study period was 11. Observed number of concussions was 2. Chi-squared contingency test, *χ*^2^ = 9.84 (*p* = 0.0017)	Study showed a statistically significant decrease in concussion rates after participating in pre-season intervention training. Note: data not specific to football players
Baker et al. (2019) [92]	*n* = 130 university hockey, football and basketball athletes (68 women, 62 men), mean age: not stated	Cohort study to explore a relationship between pre-season deep neck flexor endurance test time of uninjured athletes compared with injured athletes	Concussion was diagnosed using criteria from an inter-professional management team – 17/135 athletes sustained a concussion (6 women and 11 men). No correlation between pre-season deep neck flexor endurance test time and concussion incidence (*p* = 0.55). A moderate correlation between deep neck flexor endurance test time and concussion recovery (*r* = 0.47, *p* = 0.001)	Study found cervical endurance as measured with the deep neck flexor endurance test was not predictive of increased risk of sustaining a concussion. Note: data not specific to football players
Collins et al. (2014) [91]	*n* = 6662 high school football, basketball and lacrosse athletes (3002 girls, 3660 boys), mean age: not specified	Cohort study to determine if anthropometric measurements captured by athletic trainers can be used to predict concussion risk among high school athletes. Concussion was reported using criteria from the High-School Sports Injury Surveillance study	After adjusting for sex and sport, overall composite neck strength remained a significant predictor of concussion (*p* = 0.004) Athletes who were concussed during the season had 11–22% lower overall mean neck strength during preseason than athletes who were not concussed	Higher neck strength is associated with a lower risk of concussion in high school athletes. Note: data not specific to football players

Summary of player skill development recommendations for future heading guidelines:

- Evidence supports the importance of teaching heading technique proficiency as part of all coaching curriculums, with emphasis placed on the quality of technique training rather than focusing on higher volume of headers performed per drill or per week. Heading training could commence without ball–head contact, such as catching the ball at forehead height to teach ball tracking and body positioning.
- Given the low risk and potential high benefit associated with neck training programs, consideration should be given to incorporating neuromuscular neck exercises into general injury reduction exercise programs, such as part 2 of the FIFA 11+ or Perform+, particularly in young and adolescent athletes, who appear the most likely to benefit.

### Strategy 3: Equipment

Another strategy to mitigate heading burden is via adjustments to ball properties and personal protective equipment, particularly in young players.

#### Ball Properties

Specific ball requirements are stipulated by IFAB ‘Laws of the Game’ [89, 100]. The 2020/2021 Laws of the Game state a size 5 match ball must have a pressure equal to 8.5–15.6 pounds per square inch (psi) at sea level and weigh between 410 and 450 g (g) [100]. Smaller size 3 (311–340 g) and 4 (350–390 g) balls are recommended for younger players, with regulations usually determined by each country’s Football Association. For example, in Australia, most players will have transitioned to a size 5 ball by the time they reach 14–15 years of age. The following table (Table 5) outlines the effect that changes to ball properties, such as ball size, mass, pressure and velocity, have on head acceleration during heading. Only studies using human participants are included, although studies using headform and theoretical models demonstrate that head acceleration is influenced by a variety of factors, including speed of the ball, with higher velocity balls demonstrating higher head accelerations [101, 102]; ball pressure, with lower ball pressures having lower accelerations [16, 103]; and size, as smaller size 4 (370 g) balls were associated with lower head accelerations when compared with standard (410 g) and standard-light (350 g) size 5 balls for simulated defensive heading [104].

**Table 5:** Ball properties and their relationship with head impact magnitude

Study	Participants	Ball characteristics				Results for peak linear head acceleration (g)	Study conclusions
		Ball size	Ball mass (g)	Ball pressure (psi)	Ball velocity (m/s)		
Peek et al. (2021) [44]	*n* = 61 youth players (*n* = 35 boys), mean age: 14.52 ± 1.37 years	5 4 5 5	192 432 255 430	5 5 5 10.5	6.3	6.3 11.4 8.1 15.4	The lightest size 5 ball and the size 4 ball demonstrated linear head accelerations up to 59% lower (*p* ≤ 0.01) when compared with the size 5 higher-pressure match ball
Dorminy et al. (2015) [105]	*n* = 16 division I adult men	5	430	8	13.4 17.9 22.4	34.7 49.2 50.7	Higher ball speeds are associated with higher head acceleration
Lukášek and Kalichová (2015) [106]	*n* = 16 boys, aged 10 years	4	–	–	3.1 4.4 5.5	6.0 7.7 10.1	Results showed head accelerations increase as ball velocity increases
Funk et al. (2011) [107]	*n* = 20 healthy adults	5	430	8	5.0 8.5 10.0 11.5	6.8 15.0 18.0 21.0	Higher ball speeds are associated with higher head accelerations
Shewchenko et al. (2005) [89]	*n* = 3 healthy adult men	3 4 5 5	299 351 444 444	11.6 11.6 11.6 Various 8.7–16.0	6.0	11.9 14.3 14.5 Range 14.4–17.8	Linear acceleration decreased by 10% with a 20–32% decrease in ball mass and size. Linear acceleration decreased by 10% for a size 5 ball with a 50% reduction in ball pressure

psi = pounds per square inch, m/s = metres per second

#### Personal Protective Equipment

##### Mouth Guards

Football players are currently not required to wear mouth guards during training or games. The influence of wearing a mouth guard on head accelerations during heading in football has been reported in one study [108], as well as other studies in rugby [109] and with blows to the jaw [110]. These three studies all reported lower head accelerations with participants who used their teeth to clench the mouth guard, likely through greater engagement of their neck muscles from teeth clenching, although further investigation is required. In the only known football study to collect head acceleration data, 11 high school boys headed the ball under three different oral conditions [108]:

- drill 1, heading freely performed without instruction and without a mouth guard,
- drill 2, heading performed with the participant instructed to clench the masseter muscles tightly while not wearing the mouth guard,
- drill 3, heading performed with the participant instructed to clench tightly while wearing a mouth guard.

Weak masseter and sternocleidomastoid muscle activity was observed during drill 1, but after players were instructed to clench their masseter muscles in drills 2 and 3, statistically significant decreases in head acceleration and increases in masseter and sternocleidomastoid muscle activity were observed (*p* ≤ 0.05), with the effect stronger when players wore the mouth guard [108].

##### Head Gear

The impact between the head and a football is quite different from the impact between a head and a rigid object, due to the ability of the ball to deform when striking a harder surface [111]. With ball to head impacts, the majority of deformation occurs within the ball as it is the most compliant (softest) structure. It is surmised that the addition of a compliant headband or headgear would only change the duration of impact significantly, assuming it was as compliant as the ball and did not compress fully [111]. A soft headband of reasonable thickness would be fully compressed before the ball had completed deformation from impact, rendering it ineffective in reducing head acceleration [111]. A headband stiff enough not to compress fully could reduce peak forces and accelerations [111], but it is unclear whether these reductions would be sufficient to warrant their use. Using headform models during common football head impact scenarios, commercially available head gear did not provide substantial protection in ball-head impacts [112]. However, there was evidence of large reductions in head acceleration during head–head impacts with the magnitude of the reduction depending on the composition, thickness and coverage of the head gear, the location of the head impact and whether one or both headforms were wearing head gear [112]. In studies using human participants, mixed results are reported in youth players, with the current evidence base suggesting no effect on injury risk in adult players. A study of 278 boys and girls aged 12–17 years found players who did not wear head gear had a higher concussion rate (52.8%) than those wearing headgear (26.9%) [113]. Conversely, a' study of 2788 high school boys and girls reported that head gear did not reduce the incidence or severity of concussion [114]. In adults, it has been reported that head gear had no influence on neurocognitive performance and symptoms following an acute bout of heading in 25 varsity players aged 18–23 years [115], and that wearing head gear may increase head acceleration during heading, particularly in women, possibly due to an increase in head mass for which player neck strength is unable to compensate [50]. The use of head gear to reduce head acceleration and subsequent injury risk is likely dependent on the make and model of head gear used in each study. Therefore, further evidence is required before head gear more broadly can be recommended for football players regardless of age [4].

**Summary of Game or Team Recommendations for Future Heading Guidelines**

- During training and games, consideration should be given to using balls on the lower end of the IFAB ball pressure regulations.
- For games, a ball pressure gauge should be used to ensure match balls conform to the ball manufacturer’s pressure recommendations.
- There is very limited evidence to suggest that mouth guards may reduce head accelerations; thus further research is required before mouth guards can be recommended for universal use.
- Conflicting evidence exists regarding the protective effect of head gear and likely depends on the specific make and model of head gear worn. No recommendations regarding the use of head gear to reduce heading burden are made at this time.

## Conclusion

To mitigate the potential risk of heading in football on long-term brain health, this review has located evidence to suggest that future heading guidelines should consider the following: developing a heading coaching framework, which emphasises the technical proficiency of all aspects of heading (including, but not limited to, point of head contact, standing, running and jumping headers and body positioning for heading duels), some of which can be completed without a ball. Greater emphasis on SSGs, particularly in young players, as well as developing a rest and recovery strategy by limiting the total number of headers per week in training for players who complete higher numbers of headers in games, are important considerations. Limiting headers from goal kicks and corners, should also be considered in training (for all players) and discouraging heading from these types of ball delivery in games for young players by offering alternative approaches, such as playing out from the back or short corners, should also be considered. The inclusion of neuromuscular neck exercises integrated into general injury reduction exercise programs for all players (such as FIFA 11 +) should be further explored, as well as enforcement of rules related to deliberate head contact and ensuring that the pressure of match balls conform to IFAB regulations (with pressures at the lower end of the pressure spectrum for younger players). It is also recommended that an implementation and evaluation plan which is co-designed by health professionals, researchers, coaches, players, and other important football stakeholders is developed in tandem with any future heading guidelines to optimise the protection of brain health for all current and future players. However, despite these recommendations, it should be acknowledged that the current evidence is limited by the small sample sizes, often in singular demographics (such as boys or men, or only youth or adult players), and only using one intervention (such as head gear or neck exercises). Therefore, it is imperative that the development of any future heading guidelines is accompanied by a co-designed implementation and evaluation plan to explore whether multifaceted strategies to reduce heading burden have the desired effect in terms of translating to better long-term player outcomes.

## Data Availability

All data are contained within the paper. No datasets were generated or analysed during the current study.
